# Detroit Veterinary Medical College

**Published:** 1896-06

**Authors:** 


### COMMENCEMENT EXERCISES.

### DETROIT VETERINARY MEDICAL COLLEGE.

THE Veterinary Department of the Detroit College of Medicine held its Third Annual Commencement in the College Building, Thursday, April 2. The large lecture-hall was decorated for the occasion. The programme consisted of instrumental and vocal music, presentation of prizes, and addresses by the speakers of the evening, Dr. Charles Douglas and Hon. J. W. McGrath. Dr. H. O. Walker, Secretary of the Board of Trustees, in a few well-chosen words, conferred the degree of D.V.S. upon the successful candidates, as follows: A. J. Daiber, Detroit, Mich.; W. D. Seibert, Petoskey, Mich.; A. E. Sager, Dresden, Ontario; Z. Veldhuis, Grand Rapids, Mich.; J. E. Ward, Williamston, Mich.; J. Murray, M.D., Detroit, Mich.

The Department has bright prospects of a future of a substantial nature. A commodious new building is now under process of erection, to be completed September, 1896. The estimated cost is $30,000.

The staff of teachers for 1896–97 will consist of three having the degree of M.D.V.S., one of D.V.S.B.S., and three M.D. The management is ambitious to make the Veterinary Department of the Detroit College of Medicine a high standard college, with requirements equal to any veterinary college in America.

